# A Step in the Right Direction: Point-of-Care Ultrasound as an Evaluation Tool of Achilles Tendon Injuries in the Emergency Department

**DOI:** 10.7759/cureus.11823

**Published:** 2020-12-01

**Authors:** Chase A Silvers, Robert Lowe, Eric Cortez

**Affiliations:** 1 Emergency Medicine, OhioHealth Doctors Hospital, Columbus, USA

**Keywords:** point-of-care-ultrasound, achilles injury, obesity, lower extremity trauma, thompson test, achilles tendon

## Abstract

This report describes the diagnosis of an Achilles tendon tear in a female patient with an inconclusive physical exam, which was limited by the patient’s body habitus. Expedient use of point-of-care ultrasound supported the diagnosis of an Achilles tear with findings of a tendinous defect, fibrous stranding, and surrounding anechoic fluid, suggestive of localized hemorrhage. The patient was splinted in plantar flexion and had prompt orthopedic referral with MRI that verified Achilles tear.

## Introduction

Achilles tendon tear should be considered when evaluating traumatic lower extremity pain, as it has a 25% miss rate on initial presentation [1-2]. Clinically, injuries can be evaluated by several physical exam maneuvers, such as strength testing and the Thompson test. The Thompson test is performed with the patient lying prone with both feet off the examining table [1]. When bilateral calves are squeezed, the normal side exhibits passive plantar flexion and the injured side does not exhibit a response [1]. However, little is noted in the literature regarding applicability of these maneuvers in patients characterized as obese. In this report, physical exam findings were influenced by the patient’s body habitus and acuity of pain at presentation, demonstrating the need for further imaging. In this case, ultrasound was used as an initial imaging modality given its convenience, low cost, and lack of radiation. Point-of-care ultrasound enables visualization of Achilles injuries and involved soft tissue structures by visualizing tendinous defects, surrounding the need for further imaging [3-4]. This case discusses an obese female with inconclusive physical exam findings of Achilles injury, which was confirmed with point-of-care ultrasound at the bedside. This allowed for prompt outpatient orthopedic referral.

## Case presentation

**Figure 1:**
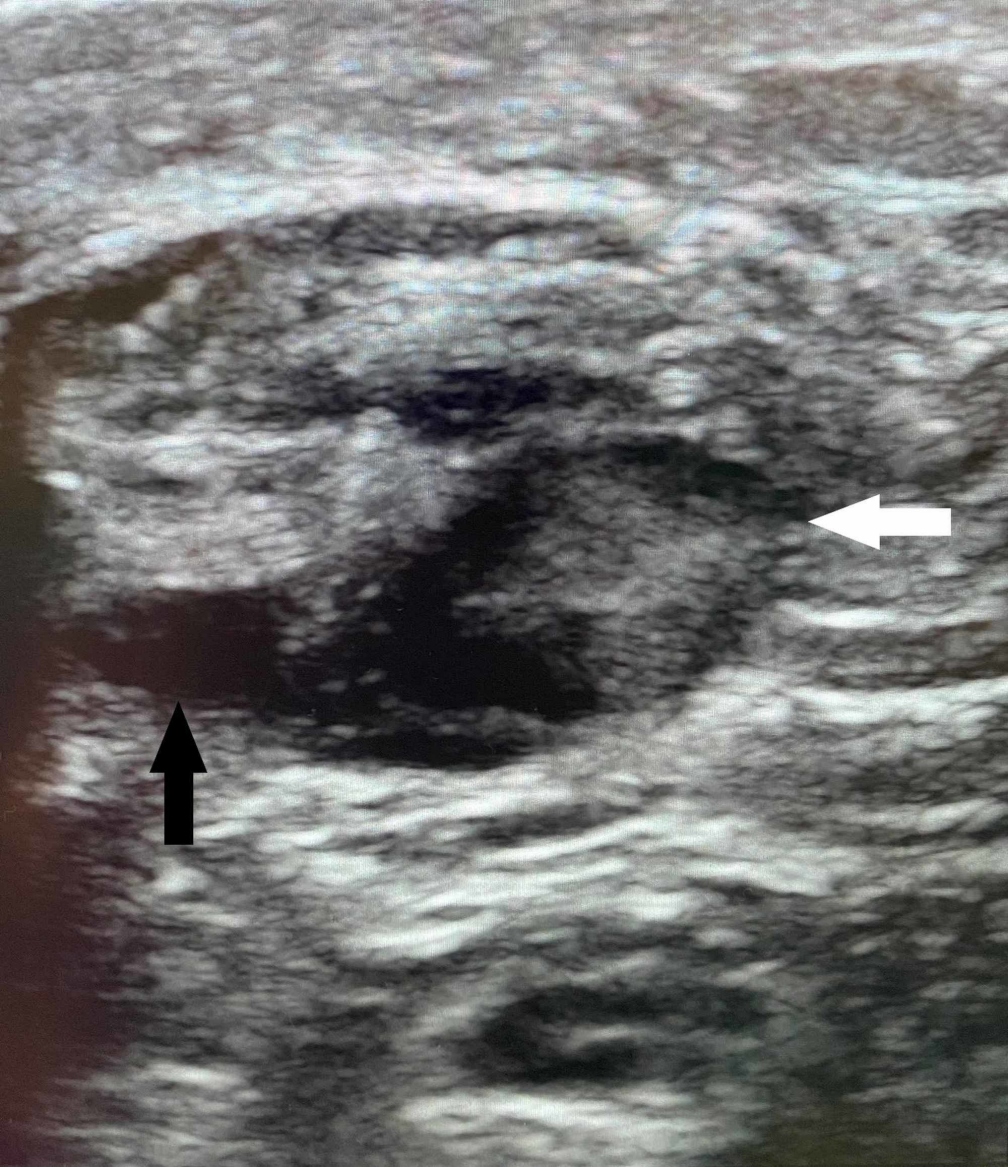
Achilles Tendon Tear in Transverse View Transverse sonographic image of Achilles tendon with fibrous disruption and surrounding anechoic fluid (white arrow) suggestive of hemorrhage. The finding of tendinous stranding and anechoic fluid suggestive of hemorrhage surrounding the gastrocnemius aponeurosis (black arrow).

**Figure 2:**
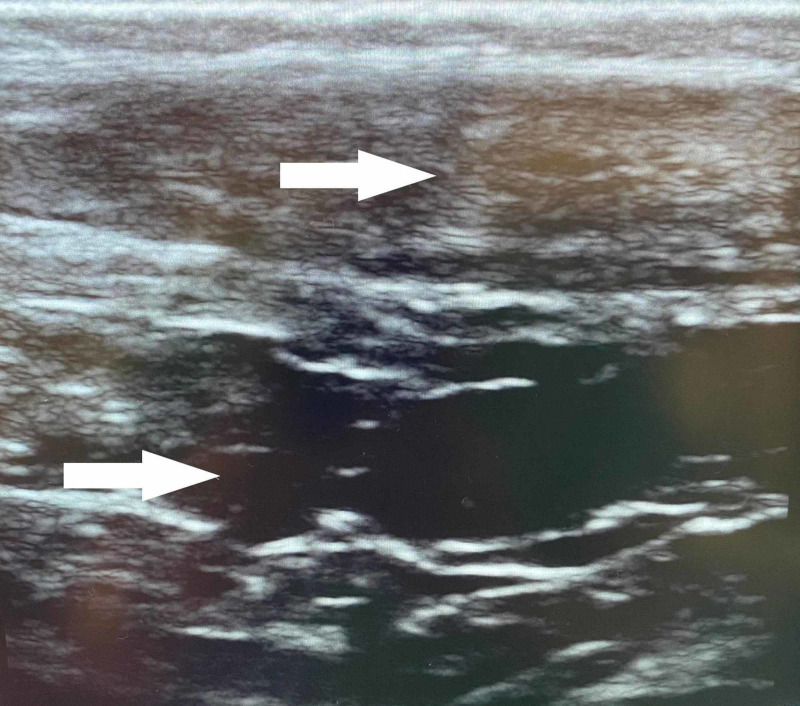
Longitudinal Ultrasound Achilles Tendon Tear Longitudinal sonographic imaging of Achilles tendon with midpoint defect (upper white arrow) with anechoic fluid posteriorly (lower white arrow) suggestive of hemorrhage and partial versus full tendinous tear.

A 46-year-old female with a history of obesity and chronic knee pain presented with the chief complaint of left leg pain. The patient suffered direct trauma to her posterior left lower extremity after a mechanical fall down stairs. She reported pain localized over the posterior left lower extremity, and was unable to ambulate secondary to pain. She reported daily use of meloxicam for chronic knee pain, and specifically denied use of corticosteroids or fluoroquinolones in the past year. Vital signs were within normal limits. She was neurovascularly intact with soft compartments. There was tenderness to palpation and mild swelling 4 cm proximal to the insertion of the calcaneus. Plantar flexion was diminished and Thompson test was performed with no plantar flexion elicited on calf squeeze. Similarly limited plantar flexion on the opposing lower extremity. These inconclusive findings were thought to limit reliability of the physical exam and believed to be secondary to patient discomfort and body habitus. Point-of-care ultrasound demonstrated an Achilles tendon defect with surrounding anechoic fluid and fibrinous stranding of tendon (Figures 1, 2). She was splinted in plantar flexion and orthopedic follow-up was arranged in two days.

## Discussion

The Achilles tendon is one of the largest and most commonly injured tendons in the human body [1]. Tears are uncommon with annual occurrences of 22-32 per 100,000 people. Most injuries result from activities performed without appropriate conditioning or warmup (“weekend warrior”) with mechanisms of forced dorsiflexion on a plantar-flexed foot or direct tendon impact causing tendinous tears and/or ultimately rupture [5]. Achilles tendon tears can be missed in up to 25% of presentations. Long-term effects of missed initial management include chronic pain, nonunion, and impaired mobility [6]. MRI is considered the gold standard for diagnosis, but is typically not feasible in the emergency department [5]. Point-of-care ultrasound (POCUS) is an efficient and less costly alternative, permitting real-time, dynamic evaluation of soft tissue injuries [5,7]. Sensitivities of clinical exam maneuvers range from 0.73-0.96 in the literature. It is unclear if certain physical traits or exam conditions (emergency department versus office) limit exam interpretation, including patients with underlying obesity which may confound exam findings [2,8-9]. Point-of-care ultrasound allows for immediate results by visualizing real-time motion and dynamics of injured soft tissues with sensitivities .94-1.00 reported [3-4, 6-7]. The clinical utility of ultrasound in Achilles tendon injuries has been previously demonstrated [10-13]. This encounter addresses the importance of point-of-care ultrasound in patients with an inconclusive physical exam limited by body habitus and acute pain. In specific cases with obese patients, point-of-care ultrasound can provide more information in a challenging patient. Further, given the risk of delayed or disrupted recovery in this patient population, especially in the setting of worsening obesity trends in the United States, it is clear that POCUS provides an opportunity to improve patient outcomes in the emergency department [14-15]. Emergency department management of Achilles tendon injuries, such as tears and ruptures, requires approximation of tendon ends with splinting in plantar flexion positioning for realignment.

## Conclusions

This patient presented with sonographic findings of a complete Achilles defect including fibrous tendon stranding and surrounding anechoic fluid suggestive of hemorrhage. Ultrasound findings obtained were uploaded and made available for the consultant at follow-up. She was splinted in plantar flexion and made non-weight bearing, with prompt referral for outpatient orthopedic evaluation two days later. This case demonstrates the clinical utility, ability to visually report findings at presentation, and an alternative means of diagnosis with difficult physical exam using point-of-care ultrasound. This allows for proper diagnosis of Achilles tendon injury, correct treatment and appropriate referral from the emergency department.

## References

[1] Lin Y, Yang L, Yin L, Duan X (2016). Surgical strategy for the chronic Achilles tendon rupture. BioMed Res Int.

[2] Maffulli N (1998). The clinical diagnosis of subcutaneous tear of the Achilles tendon. A prospective study in 174 patients. Am J Sports Med.

[3] Dong Q, Fessell DP (2009). Achilles tendon ultrasound technique. Am J Roentgenol.

[4] Hartgerink P, Fessell DP, Jacobson JA, van Holsbeeck MT (2001). Full- versus partial-thickness Achilles tendon tears: sonographic accuracy and characterization in 26 cases with surgical correlation. Radiology.

[5] Gulati V, Jaggard M, Said Al-Nammari S (2015). Management of Achilles tendon injury: a current concepts systematic review. World J Orthop.

[6] Liu W, Zhuang H, Shao D, Wang L, Shi M (2017). High-frequency color Doppler ultrasound in diagnosis, treatment, and rehabilitation of Achilles tendon injury. Med Sci Monit.

[7] Poposka A, Georgieva D, Dzoleva-Tolevska R (2012). Significance of ultrasound in the diagnosis and treatment of Achilles tendon rupture. Prilozi.

[8] Kälebo P, Allenmark C, Peterson L, Swärd L (1992). Diagnostic value of ultrasonography in partial ruptures of the Achilles tendon. Am J Sports Med.

[9] Silk AW, McTigue KM (2011). Reexamining the physical examination for obese patients. Jama.

[10] Adhikari S, Marx J, Crum T (2012). Point-of-care ultrasound diagnosis of acute Achilles tendon rupture in the ED. Am J Emerg Med.

[11] Lee W-J, Tsai W-S, Wu R-H (2013). Focused ultrasound for traumatic ankle pain in the emergency department. J Emerg Med.

[12] Odom M, Haas N, Phillips K (2018). Bedside ultrasound diagnosis of complete Achilles tendon tear in a 25-year-old man with calf injury. J Emerg Med.

[13] Stickles SP, Friedman L, Demarest M, Raio C (2015). Achilles tendon rupture. West J Emerg Med.

[14] American Academy of Orthopaedic Surgeons (2015). Position Statement: The Impact of Obesity on Bone and Joint Health.

[15] Flegal KM, Carroll MD, Ogden CL, Curtin LR (2010). Prevalence and trends in obesity among US adults, 1999-2008. JAMA.

